# Protective effect of β-sitosterol against high-fructose diet-induced oxidative stress, and hepatorenal derangements in growing female sprague-dawley rats

**DOI:** 10.1186/s42826-024-00215-5

**Published:** 2024-08-26

**Authors:** Nontobeko M. Gumede, Busisani W. Lembede, Pilani Nkomozepi, Richard L. Brooksbank, Kennedy H. Erlwanger, Eliton Chivandi

**Affiliations:** 1 Department of Physiology, School of Medicine, Faculty of Health Sciences, University of Pretoria, Private bag X32, Pretoria, 0031 South Africa; 2 School of Physiology, Faculty of Health Sciences, University of the Witwatersrand, 7 York Road, Parktown, Johannesburg, 2193 Republic of South Africa; 3 Department of Human Anatomy and Physiology, Faculty of Health Sciences, University of Johannesburg, Doornfontein, Johannesburg, Republic of South Africa

**Keywords:** Renal injury, Non-alcoholic fatty disease, Β-sitosterol, High-fructose diet, Oxidative stress

## Abstract

**Background:**

Chronic consumption of a high-fructose diet causes oxidative stress that compromises kidney and liver health. β-sitosterol (Bst), a phytosterol, is a functional nutrient with health benefits. β-sitosterol antioxidant activity protects the liver and kidney from ROS-mediated damage and lipid peroxidation. We evaluated the potential renoprotective and hepatoprotective effects of orally administrated β-sitosterol in high-fructose diet-fed growing female rats. Thirty-five 21-day old female Sprague-Dawley rat pups were randomly assigned to and administered the following treatments for 12 weeks: group I- standard rat chow (SRC) + plain drinking water (PW) + plain gelatine cube (PC); group II- SRC + 20% w/v fructose solution (FS) as drinking fluid + PC; group III- SRC + FS + 100 mg/kg body mass (BM) fenofibrate in gelatine cube; group IV- SRC + FS + 20 mg/kg BM β-sitosterol gelatine cube (Bst) and group V- SRC + PW + Bst. The rats were fasted overnight, weighed then euthanised. Blood was collected, centrifuged and plasma harvested. Livers and kidneys were excised, weighed and samples preserved for histological assessments. Plasma biomarkers of oxidative stress, liver and kidney function and renal tubular injury were assessed.

**Results:**

High fructose diet fed rats had increased plasma KIM-1, NGAL (*p* < 0.001) and MDA levels (*p* < 0.05). Dietary fructose caused microvesicular and macrovesicular steatosis, and reduced glomerular density, Bowman’s capsule area and urinary space. β-sitosterol protected against the high-fructose diet-induced hepatic steatosis and glomerular disturbances without adverse effects on liver and kidney function.

**Conclusions:**

β-sitosterol, as a dietary supplement, could potentially be exploited to prevent high-fructose diet-induced NAFLD and to protect against high-fructose diet-induced renal tubular injury.

## Background

Non-communicable chronic diseases (NCDs) are the leading cause of deaths worldwide. According to the World Health Organisation (WHO), NCD accounts for 74% of all deaths globally. Physical inactivity, smoking and the consumption of unhealthy diets are the main risk factors for the development of NCDs [1]. Globally, there is a high use of fructose as a sweetener in foods and beverages [2]. The daily dietary fructose intake increased by 5-fold from the 1950s to 2000, concomitant with this, the increase in prevalence of obesity and metabolic syndrome [3, 4]. Fructose metabolism by-passes the rate-limiting step of glycolysis making it highly lipogenic [5]. In addition to mediating increased lipogenesis, excessive fructose intake causes dyslipidemia, visceral adiposity and non-alcoholic fatty liver disease (NAFLD) [6]. NAFLD, a leading cause of liver disease, is considered a hepatic manifestation of metabolic syndrome [6]. The global prevalence of NAFLD has been estimated to be over 25% [7]. It is expected to increase with an estimated 3.6 million new cases per year [7]. The early stage of NAFLD is simple non-alcoholic fatty liver (NAFL) characterised by hepatic steatosis. However, NAFLD can progress to non-alcoholic steatohepatitis (NASH), characterized by the signs of hepatic inflammation, which can progress to liver fibrosis and cirrhosis [8].

Further to causing the development and progression of NAFLD, the excessive intake of dietary fructose has been shown to induce structural and functional alterations in the kidneys, elevating the risk of developing chronic kidney disease (CKD) [9]. According to the Global Burden of Disease, kidney disease is the leading cause of worldwide mortality [10]. CKD has a high prevalence with over 697.5 million cases reported worldwide in 2017 and is predicted to become the fifth leading cause of death by 2040 [10, 11]. Recent epidemiological trends have indicated that the global prevalence of CKD is 10% of the world population affecting > 800 million people [12]. Histopathological analysis of renal tissues exposes early changes that could indicate the onset of kidney disease [13]. Thus, histological assessments have, traditionally, been regarded as the gold standard for investigating renal function [13]. In addition, neutrophil gelatinase-associated lipocalin (NGAL) and kidney injury molecule-1 (KIM-1), have recently attracted much attention as useful biomarkers for assessing renal tubular injury [14].

Lifestyle modification and the use of various synthetic pharmaceutical agents are employed to manage the components of metabolic syndrome, NAFLD, and kidney disease [15]. However, synthetic pharmaceutical drugs are associated with increased side effects, are generally inaccessible, expensive and mono-therapeutic [16] hence the increased use of plant-derived ethnomedicines. β-sitosterol (Bst), the most abundant of phytosterols, is reported to exert anti-obesogenic, anti-diabetic and anti-inflammatory activities [17]. In view of the increase in diet-induced kidney and liver disturbances, there is a dire need to explore preventative interventions early in life. Considering the multiple health beneficial biological activities of β-sitosterol, we aimed to evaluate the prophylactic effect of β-sitosterol against the development of diet-induced renal and liver disease in a rat experimental model.

## Methods

The study was performed in accordance with ARRIVE guidelines [18].

### Study site

The study was conducted at the Wits Research Animal Facility of the University of the Witwatersrand, Johannesburg, South Africa. Ethical clearance was granted by the Animal Ethics Screening Committee (AESC) of the University of Witwatersrand (AESC clearance number 2017/08/55/B). Compliance with accepted laboratory animal use and care stipulated in the South African National Standard (SANS: 10386:2008) and the animal protection act 1962, Act No. 71 was adhered to during the course of the study.

### Chemicals and reagents

All the chemicals and reagents used in the study were of analytical grade. β-sitosterol, fenofibrate and dimethyl sulfoxide (DMSO) were purchased from Sigma-Aldrich (St. Louis, Missouri, USA). Fructose was obtained from Nature’s Choice (Randvaal, South Africa).

### Experimental animals and management

The study used thirty-five 21-day old female Sprague-Dawley rat pups. The rat pups were habituated to handling for two days (postnatal days 21 and 22) prior to the commencement of the experiment on postnatal day 23. The rats were housed individually in acrylic cages, with wood shavings for bedding. They were allowed *ad libitum* access to a standard rat chow (Nutritionhub (PTY) Ltd, Stellenbosch, South Africa) and drinking fluid; water and/ or 20% fructose solution. The rat pups were housed in Perspex cages which were lined with wood shavings and shredded paper for environmental enhancement. A twelve-hour light and dark cycle were followed with lights on from 7 am to 7 pm. Room temperature was maintained at 26 ± 2^°^C throughout the experimental period.

### Experimental design

On post-natal (PND) day 23 the thirty-five female pups were randomly distributed to and fed the following treatment regimens for 12 weeks: group 1 (negative control): standard rat chow (SRC) + plain drinking water (PW) + plain gelatine cube (PC), group 2 (metabolic dysfunction induced): SRC + 20% w/v fructose solution (FS) to drink + PC, group 3 (positive control) SRC + FS + 100 mg/kg body mass per day of fenofibrate in a gelatine cube (FF), group 4 (potential prophylactic effects of Bst): SRC + FS + 20 mg/kg body mass per day of β-sitosterol in a gelatine cube and group 5 (impact of Bst alone): SRC + PW + Bst. Our choice of fenofibrate and β-sitosterol dosages were as recommended by Baskar et al., 2010 [19] and by Ji et al., 2005 [20], respectively. Throughout the experiment, the rats were weighed twice weekly in order to monitor growth and general health and for the adjustment of the dosage of the treatments considering body mass changes over the 12-week period.

### Terminal procedures and computations

At the end of the 12-week experimental period, the rats were fasted overnight but allowed access to plain drinking water to prevent dehydration. The rats were euthanised with an intraperitoneal injection of sodium pentobarbitone at 200 mg/kg body mass. Following euthanasia, blood was collected via cardiac puncture using 18G needles and 10 ml syringes, into heparinised blood collection tubes (Becton Dickinson Vacutainer Systems Europe, Meylan Cedex, France). Immediately after, the blood samples were centrifuged at 20^○^C for 15 min at 5000 × g in a Sorvall RT^®^ 6000B centrifuge (Pegasus Scientific Inc., Rockville USA). The plasma was then decanted into 1.5 ml microtubes and frozen-stored in a freezer at -80˚C pending determination of oxidative stress, antioxidant status and surrogate markers of liver and kidney function. Following blood sample collection, the abdomen was cut via a midline incision and the kidneys and liver were dissected out. Each rat’s liver and kidneys were weighed on an electronic scale (Presica 310 M, Presica Instruments, Dietikon, Switzerland). The liver was then divided into two parts: one fixed in 10% phosphate-buffered formalin solution (Merck, Johannesburg, South Africa) for histological analysis and the other was stored in sealed ziplock plastic bag at -20 °C for total fat content determination. The right kidney from each rat was preserved in 10% phosphate-buffered formalin for histology analysis.

The kidney and liver mass relative to body mass (%BM) was computed by dividing the mass of each organ (liver and or/kidney) by the respective terminal body mass of each rat and expressed as a percentage.

Relative kidney or liver mass = [organ mass (g) /terminal body mass (g)] x 100.

### Determination of liver lipid content

The total liver lipid content was determined using the Soxhlet extraction method as described by the Association of Analytical Chemists [AOAC method number 920.39] using petroleum ether as the solvent.

### Lipid peroxidation assay

Plasma TBARS concentration was estimated using the method described by Rakita, et al. [21]. Briefly thiobarbituric acid (TBA) standard stock solution was prepared by adding thiobarbituric acid (TBA) powder, with 60 mL of glacial acetic acid and 60 mL double distilled water. 0.2 mL of plasma sample were mixed with 0.4 mL of chromogenic agent solution and 0.2 mL of acid reagent. This mixture was placed in 10 ml glass tubes and incubated in the water bath at 100 °C for 1 h followed by centrifugation at 1600 × g for 10 min. The supernatants were pipetted into the microplate and read at 532 nm with a microplate reader (Bio-Tek Instruments, Vermont, USA). Results were expressed as ${\rm{\mu mol/L}}$. The TBARS concentration in each sample was computed using the formula:

$${\rm{TBARS}}\,{\rm{(\mu mol/L) = (\Delta A - b) \div a}}\,{\rm{x}}\,{\rm{f}}$$

where ∆A is the Absolute OD (OD_Plasma_ – OD_Blank_), a and b are the slope and intercept of standard curve, respectively, and f is the dilution factor of sample before test.

### Total antioxidant capacity T-AOC (ABTS, enzyme method)

Total antioxidant capacity was performed using the method described by Benzie and Strain, 1996. ABTS working solution was prepared according to the ratio (reagent 1: reagent 2: reagent 3 = 152:10:8). Reagent 1 consisted of buffer solution; reagent 2 consisted of ABTS solution; reagent 3 consisted of H_2_0_2_ solution mixed with double distilled water. 10 µL plasma samples were mixed with 20 µL peroxidase and 170 µL ABTS working solution. The mixture was allowed to stand for 6 min at room temperature and the absorbance was read at wavelength of 414 nm using a spectrophotometer.

Total antioxidant capacity (T-AOC) were expressed as mmol/L. Total antioxidant capacity in each sample was computed using the formula:

$${\rm{T - AOC }}\left( {{\rm{mmol/L}}} \right){\rm{ = }}\left( {{{\rm{A}}_{{\rm{414}}}}{\rm{ - b}}} \right){\rm{ \div a x f}}$$

### Determination of surrogate plasma markers of kidney and liver function

The effect of treatment on markers of liver function, namely aspartate aminotransferase (AST) and alanine aminotransferase (ALT), and markers of renal function, specifically creatinine and urea, were determined using a colorimetric-based clinical chemistry analyser (IDEXX VetTest^®^ Clinical Chemistry Analyser, IDEXX Laboratories Inc., USA) as per the manufacturer’s instructions. AST and ALT activities, creatinine and urea concentrations were reported as U/L, µmol/L, and mmol/L respectively.

### Determination of renal tubular injury markers

Plasma kidney injury molecule-1 (KIM-1), and neutrophil gelatinase-associated lipocalin (NGAL-1) concentrations were measured using a rat ELISA kit (Rat KIM-1(Kidney Injury Molecule 1) ELISA Kit, Houston, TX, USA) and rat NGAL-1 ELISA kit, (Elabscience^®^ Rat NGAL (Neutrophil Gelatinase Associated Lipocalin) ELISA Kit, Houston, TX, USA) respectively. A standard curve was constructed using known concentrations. The concentrations of KIM-1 and NGAL-1 in the samples were then determined from the respective constructed standard curve.

### Histomorphometry of the kidneys

The kidney samples were routinely processed using an automatic tissue processor (Micron STP 120, Thermo Fischer Scientific, USA), embedded in paraffin wax, sectioned at 5 μm and stained with haematoxylin and eosin (H&E) [22]. Triplicate digital images of the kidney sections were obtained from different fields using a camera (AxioCam ERc 5s Rev.2; ZEISS, Germany) connected to a light microscope. Bowman’s space area, glomerular tuft and glomerular density were obtained using a computerised morphometric analysis system (ImageJ 1.47v, Java 1.8.0_191; LOCI, University of Wisconsin). Urinary space and glomerular density were computed according to the following formulas:

Urinary space = Bowman’s space area (µm^2^) - glomerular tuft area (µm^2^) [23].

Glomerular density = number of glomerular corpuscles in a section (N)/ total area of the section (µm^2^).

The assessment of the images was done with blinding of the groups to avoid bias.

### Liver histology and scoring for non-alcoholic fatty liver disease

Following fixation, the liver tissues were routinely processed, embedded in paraffin wax, sectioned at 5 μm and stained with haematoxylin and eosin (H&E) [22]. To assess the hepatocellular changes, three random fields per slide were viewed under a light microscope at low and high-power magnification. To assess the severity and progression of fatty liver disease, the semi-quantitative NAFLD activity score (NAS) method was used [24, 25]. Macrosteatosis and microsteatosis grade scoring was as follows: Grade 0: 0–5%; Grade 1:5–33%; Grade 2: 33–66%; Grade 3; > 66%. In addition, inflammation was graded as follows: Grade 0: none or no foci of inflammation per camera field; Grade 1: 1–2; Grade 2: >2 foci per camera field. Hypertrophy/ballooning scoring: Grade 0: none; Grade 1: 1–2; Grade 2: >2. The total NAS score interpretation was scored < 2 = not steatohepatitis; 3–4 = uncertain; ≥ 5 = steatohepatitis. The scoring was done with the observer blinded to the experimental groups.

### Statistical analysis

GraphPad Prism 6.0 (Graph-pad Software Inc. San Diego, USA) software was used to analyse data. Continuous data are expressed as mean ± SD. Multiple-group normally distributed data were analysed using a parametric test, one-way ANOVA, followed by Tukey’s *post-hoc* test. The Kruskal–Wallis test was used to analyse multiple group non-normally distributed data, followed by the Dunns post hoc test. Significance was accepted when *p* < 0.05.

## Results

### Body mass

Table 1 presents the rats initial and terminal body masses. The initial body masses of the rats were similar (*p* > 0.05). Following the 12-week intervention, the rats across treatments significantly grew (*p* < 0.0001; initial body masses compared to terminal body masses). The rats’ terminal body masses were similar (*p* > 0.05) across treatments.

**Table 1:** Growth pattern comparison across experimental groups with high fructose and β-sitosterol treatment of female rats

Parameter	PC + PW	PC + FS	FF + FS	Bst + PW	Bst + FS
Initial body mass (g)	40.85 ± 4.56^a^	44.83 ± 4.39^a^	38.85 ± 4.06^a^	36.72 ± 3.20^a^	37.57 ± 2.82^a^
Terminal body mass (g)	251.71 ± 14.38^b^	263.33 ± 10.98^b^	262.71 ± 10.98^b^	256.71 ±48.66^b^	269.29 ± 54.84^b^

abWithin column and row means with different superscripts are significantly different at *p* < 0.0001. PC + PW = plain gelatine cubes + plain tap water to drink; PC + FS = plain gelatine cubes + 20% (w/v) fructose solution to drink; FF + FS = gelatine cube containing fenofibrate at a dose of 100 mg/kg body mass per day + 20% (w/v) fructose solution to drink. Bst + PW = gelatine cube containing β-sitosterol at a dose of 20 mg/kg body mass per day + plain tap water to drink; Bst + FS = gelatine cube containing β-sitosterol at a dose of 20 mg/kg body mass per day + 20% (w/v) fructose solution to drink. Data presented as mean ± SD; *n* = 7 per treatment

### Liver and kidney masses

Table 2 presents the liver and kidney masses of the rats following treatment administration for 12 weeks. The high-fructose diet had no effect (*p* > 0.05) on rats’ absolute and relative liver masses when compared to control, β-sitosterol and β-sitosterol + fructose groups. However, rats fed the high-fructose diet and administered fenofibrate had significantly heavier (*p* < 0.05) absolute and relative liver masses compared to all counterparts. There were no significant differences (*P* > 0.05) in both the absolute and relative kidney masses across the treatment regimens.

**Table 2:** The effect of β-sitosterol on absolute and relative (to body mass) liver and kidney masses of growing female rats fed a high-fructose diet

Parameter	PC + PW	PC + FS	FF + FS	Bst + PW	Bst + FS	Significance
Liver (g)	6.53 ± 0.91^ab^	7.35 ± 0.54^abc^	8.08 ± 0.67^c^	6.26 ± 0.54^a^	6.99 ± 0.48^a^	*
Liver (%BM)	2.64 ± 0.27^ab^	2.84 ± 0.14^ab^	3.26 ± 0.31^c^	2.50 ± 0.18^a^	2.82 ± 0.24^a^	*
Kidney (g)	1.43 ± 0.12^a^	1.60 ± 0.08^a^	1.56 ± 0.17^a^	1.47 ± 0.12^a^	1.44 ± 0.21^a^	ns
Kidney (%BM)	0.58 ± 0.05^a^	0.62 ± 0.03^a^	0.63 ± 0.01^a^	0.59 ± 0.05^a^	0.58 ± 0.08^a^	ns

abcWithin row means with different superscripts are significantly different at *p* < 0.05. ns = not significant; * *p* < 0.05. PC + PW = plain gelatine cubes + plain tap water to drink; PC + FS = plain gelatine cubes + 20% (w/v) fructose solution to drink; FF + FS = gelatine cube containing fenofibrate at a dose of 100 mg/kg body mass per day + fructose solution to drink. Bst + PW = gelatine cube containing β-sitosterol at a dose of 20 mg/kg body mass per day + plain tap water to drink; Bst + FS = gelatine cube containing β-sitosterol at a dose of 20 mg/kg body mass per day + fructose solution to drink (w/v); %BM = relative to body mass; Data presented as mean ± SD; *n* = 7 per treatment

### Liver lipid content

**Fig. 1:**
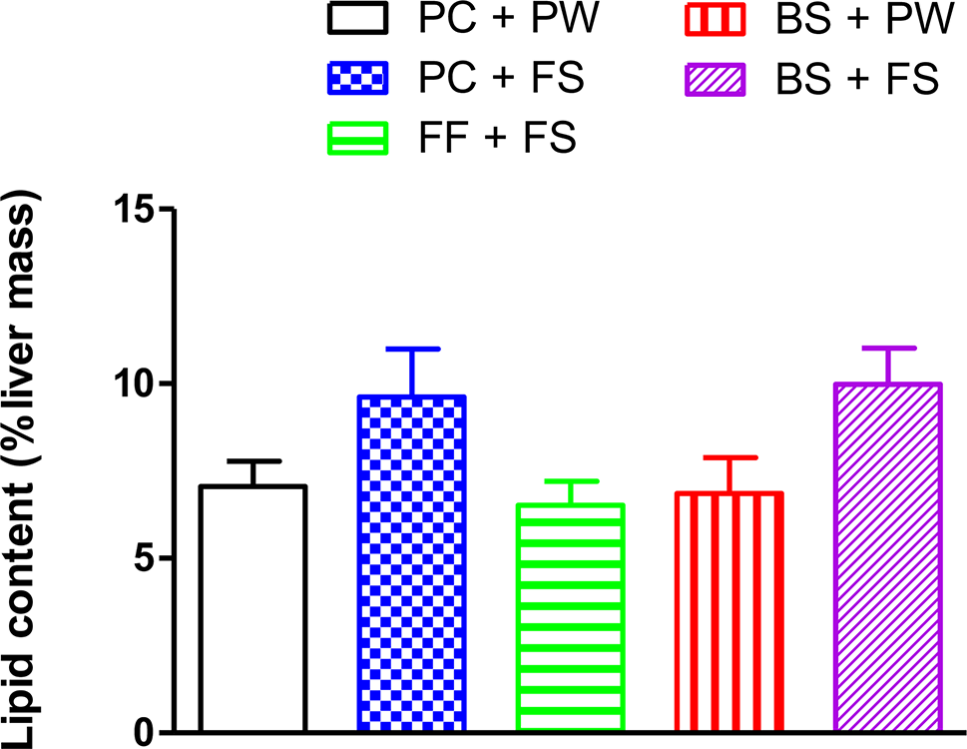
Effect of β-sitosterol on the liver lipid content of high-fructose diet-fed growing female Sprague-Dawley rats. PC + PW = plain gelatine cubes + plain tap water to drink; PC + FS = plain gelatine cubes + 20% (w/v) fructose solution to drink; FF + FS = gelatine cube containing fenofibrate at a dose of 100 mg/kg body mass per day + fructose solution to drink. Bst + PW = gelatine cube containing β-sitosterol at a dose of 20 mg/kg body mass per day + plain tap water to drink; Bst + FS = gelatine cube containing β-sitosterol at a dose of 20 mg/kg body mass per day + fructose solution to drink (w/v). Data presented as mean ± SD; *n* = 7 per treatment regimen.

Figure 1 shows the total liver lipid content of the rats following treatment administration for 12 weeks. Although there was no statistically significant difference in the rats’ liver lipid content (*P* > 0.05), rats fed the high fructose diet (PC + FS) had a 25% increase in total liver lipid content compared to control counterparts.

### Surrogate markers of liver and kidney function

Table 3 show plasma activities and concentrations of surrogate markers of liver and kidney function of the rats following treatment administration for 12 weeks. There were no significant differences (*p* > 0.05; Table 3) in the ALT and AST activities and the urea and creatinine concentrations across treatment groups.

**Table 3:** Effect of β-sitosterol on biomarkers of renal and hepatic function, in growing female rats fed a high-fructose diet

Parameter	PC + PW	PC + FS	FF + FS	Bst + PW	Bst + FS	Significance
ALT (U/L)	45.85 ± 11.09^a^	46.71 ± 9.44^a^	63.57 ± 16.46^a^	57.66 ± 10.13^a^	63.14 ± 22.45^a^	ns
AST (U/L)	157.14 ± 51.88^a^	172 00 ± 22.12^a^	183.14 ± 23.78^a^	172.16 ± 41.02^a^	189.57 ± 50.93^a^	ns
Creatinine (μmol/L)	20.37 ± 4.22^a^	23.99 ± 4.31^a^	25.26 ± 3.34^a^	24.66 ± 4.56^a^	23.99 ± 4.31^a^	ns
Urea (mmol/L)	6.78 ± 1.03^a^	6.32 ± 1.18^a^	6.88 ± 1.23^a^	6.55 ± 0.86^a^	5.92 ± 1.22^a^	ns

aWithin row means are similar at *p* > 0.05; ns = not significant, *p* > 0.05. PC + PW = plain gelatine cubes + plain tap water to drink; PC + FS = plain gelatine cubes + 20% (w/v) fructose solution to drink; FF + FS = gelatine cube containing fenofibrate at a dose of 100 mg/kg body mass per day + fructose solution to drink. Bst + PW = gelatine cube containing β-sitosterol at a dose of 20 mg/kg body mass per day (Bst) + plain tap water to drink; Bst + FS = gelatine cube containing β-sitosterol at a dose of 20 mg/kg body mass per day + fructose solution to drink (w/v); AST = aspartate aminotransferase; ALT = alanine aminotransferase. Data presented as mean ± SD; *n* = 7 per treatment

### Renal tubular injury markers

Table 4 shows plasma concentration of KIM-1 and NGAL. KIM-1 was too low to be detected in control group. The high-fructose diet increased (*p* < 0.0001) plasma concentration of KIM-1 and NGAL. Both β-sitosterol (Bst + FS) and fenofibrate (FF + FS) failed to protect against the high-fructose diet-induced increase in KIM-1 and NGAL (*p* > 0.05). β-sitosterol (Bst + PW) alone increased KIM-1 and NGAL (*p* < 0.0001) compared to control.

**Table 4:** Effect of β-sitosterol on kidney injury molecule-1 and neutrophil gelatinase-associated lipocalin in growing female rats fed a high-fructose diet

Parameter	PC + PW	PC + FS	FF + FS	Bst + PW	Bst + FS	Significance
KIM-1 (pg/mL)	N.D	143.55 ± 69.31^b^	162.35 ± 52.97^b^	180.91 ± 0.55^b^	180.55 ± 0.24^b^	*
NGAL (pg/mL)	0.13 ± 0.09^a^	2.13 ± 0.55^b^	2.13 ± 0.20^b^	1.83 ± 0.10^b^	2.06 ± 0.10^b^	***

abWithin row means with different superscripts are significantly different at *p* < 0.05. * *p* < 0.05; *** *p* < 0.001. PC + PW = plain gelatine cubes + plain tap water to drink; PC + FS = plain gelatine cubes + 20% (w/v) fructose solution to drink; FF + FS = gelatine cube containing fenofibrate at a dose of 100 mg/kg body mass per day + fructose solution to drink. Bst + PW = gelatine cube containing β-sitosterol at a dose of 20 mg/kg body mass per day (Bst) + plain tap water to drink; Bst + FS = gelatine cube containing β-sitosterol at a dose of 20 mg/kg body mass per day + fructose solution to drink (w/v); N.D = not detected; KIM-1 = kidney injury molecule-1; NGAL = neutrophil gelatinase associated lipocalin. Data presented as mean ± SD; *n* = 4–7 per treatmen

### Lipid peroxidation and antioxidant status

Table 5 shows the effects of high-fructose diet and treatments on malondialdehyde (MDA) concentration using TBARS assay and total antioxidant capacity (T-AOC). Consumption of the high-fructose diet (PC + FS) resulted in a significant increase in the level of MDA when compared to the control group (*p* < 0.05). However, the MDA concentration of the PC + PW, FF + FS, Bst + FS and Bst + PW was similar (*P* > 0.05). The T-AOC in high fructose-fed rats was significantly lower when compared control (*p* < 0.05). The decrease in total antioxidant capacity levels due to high-fructose administration was attenuated by the administration of β-sitosterol (Bst + FS) and fenofibrate (*p* < 0.05). Administration of β-sitosterol (Bst + PW) alone significantly increased T-AOC when compared to control group (*p* < 0.0001).

**Table 5:** Effect of β-sitosterol on oxidative stress and total antioxidants concentration of growing female rats fed a high-fructose diet

Parameter	PC + PW	PC + FS	FF + FS	Bst + PW	Bst + FS	Significance
TBARS (µmol/L)	14.68 ± 3.09^a^	24.02 ± 4.33^b^	17.74 ± 3.45^ab^	20.17 ± 3.40^ab^	18.61 ± 4.37^ab^	**
T-AOC (mmol/L)	1125.53 ± 49.95^a^	1049.30 ± 55.25^b^	1133.40 ± 36.71^a^	1217.56 ± 54.70^c^	1189.03 ± 42.99^ac^	***

abcWithin row means with different superscripts are significantly different at *p* < 0.05.; ** *p* < 0.05; *** *p* < 0.0001. PC + PW = plain gelatine cubes + plain tap water to drink; PC + FS = plain gelatine cubes + 20% (w/v) fructose solution to drink; FF + FS = gelatine cube containing fenofibrate at a dose of 100 mg/kg body mass per day + fructose solution to drink. Bst + PW = gelatine cube containing β-sitosterol at a dose of 20 mg/kg body mass per day + plain tap water to drink; Bst + FS = gelatine cube containing β-sitosterol at a dose of 20 mg/kg body mass per day + fructose solution to drink (w/v); TBARS = thiobarbituric acid reactive substances; T-AOC = total antioxidant capacity. Data presented as mean ± SD; *n* = 7 per treatment

### Non-alcoholic fatty liver disease activity score

Table 6 depicts the micro- and macro-vesicular, hypertrophy, lobular inflammation scores and total NAS score of the rats across treatments. The high-fructose diet (PC + FS) caused micro- and macro-vesicular hepatic steatosis when compared to PC + PW (Table 6). β-sitosterol (Bst + FS) and fenofibrate (FF + FS) attenuated the high-fructose diet-induced micro-steatosis and prevented (*p* < 0.01) macro- hepatic steatosis in (*p* = 0.0063) when compared to PC + FS.

**Table 6:** Effect of β-sitosterol on non-alcoholic fatty liver disease activity score in the liver of female rats fed a high fructose diet

Criteria	PC + PW	PC + FS	FF + FS	Bst + PW	Bst + FS	Significance
Micro-steatosis	0 (0, 0)^a^	2 (1, 2)^b^	1 (0, 1)^ab^	0 (0, 0)^a^	1 (1, 1)^ab^	*
Macro-steatosis	0 (0, 0)^a^	2 (1, 3)^b^	0 (0, 0)^ab^	0 (0, 0)^a^	0 (0, 0)^a^	**
Hypertrophy	0 (0, 1)^a^	2 (0, 2)^a^	0 (0, 1)^a^	0 (0, 0)^a^	0 (0, 1)^a^	ns
Inflammation	0.5 (0, 1)^a^	2 (1, 2)^a^	0 (0, 1)^a^	0.5 (0, 1)^a^	1 (0, 1)^a^	ns
Total NAS	0.5 (0, 2)^a^	3.5 (2, 6)^a^	1.5 (0, 3)^a^	0.5 (0, 1)^a^	2 (2, 2)^a^	ns

ab Within row means with different superscripts are significantly different at *p* < 0.05.*p < 0.05; **p < 0.001. ns = not significant, *p* > 0.05. PC + PW = plain gelatine cubes + plain tap water to drink; PC + FS = plain gelatine cubes + 20% (w/v) fructose solution to drink; FF + FS = gelatine cube containing fenofibrate at a dose of 100 mg/kg body mass per day + fructose solution to drink. Bst + PW = gelatine cube containing β-sitosterol at a dose of 20 mg/kg body mass per day + plain tap water to drink; Bst + FS = gelatine cube containing β-sitosterol at a dose of 20 mg/kg body mass per day + fructose solution to drink (w/v); NAS = non-alcoholic fatty liver disease activity score. Data presented as median and range (min, max). *n* = 4 per treatment. Total NAS score interpretation: <2 = not steatohepatitis; 3–4 = uncertain; ≥ 5 = steatohepatitis

### Liver histomorphometry

**Fig. 2:**
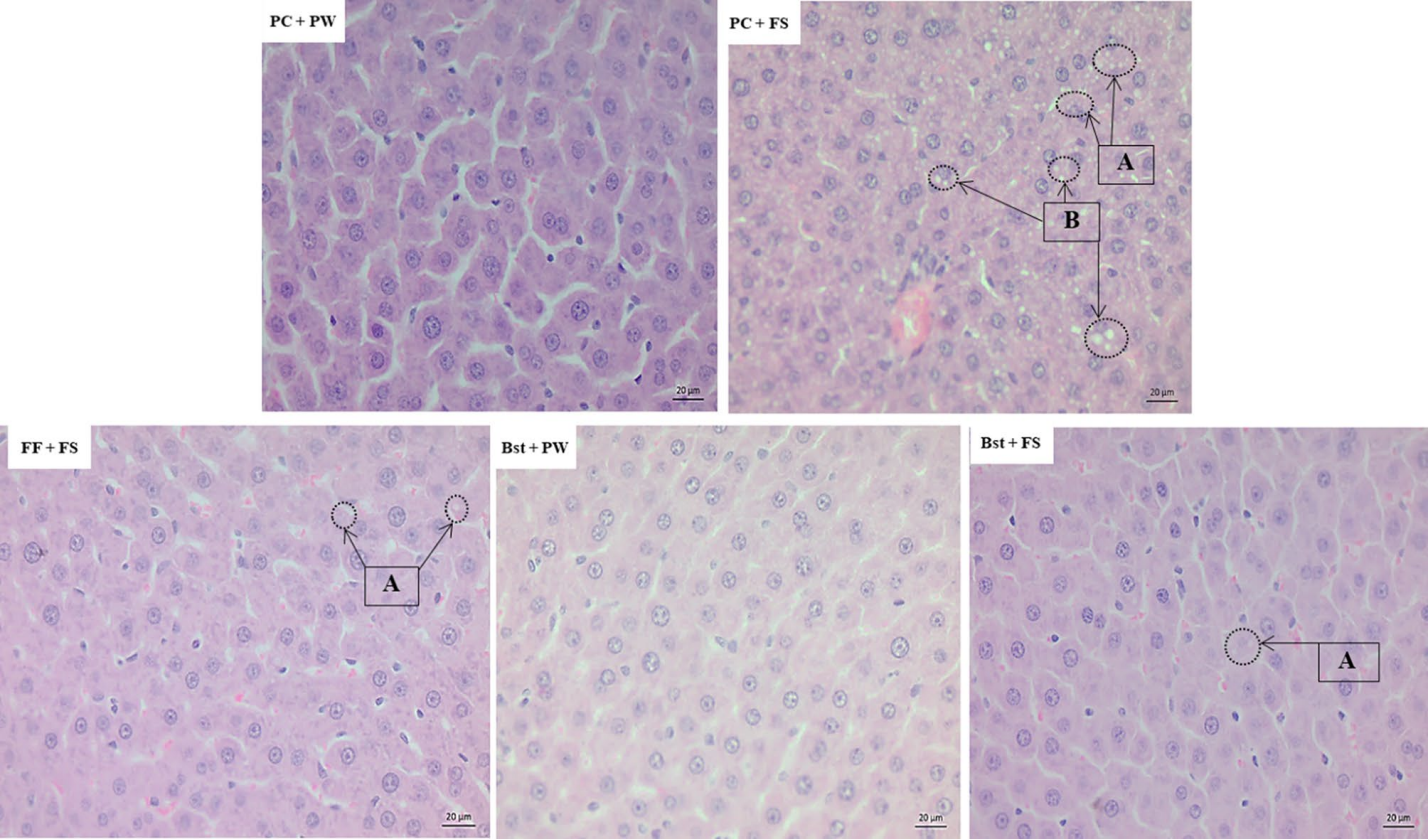
The effect of β-sitosterol on liver histopathological analyses (H and E staining, 40 X magnification). Arrows A point to hepatic micro-vesicular steatosis, arrows B point to macro-vesicular steatosis. PC + PW = plain gelatine cubes + plain tap water to drink; PC + FS = plain gelatine cubes + 20% (w/v) fructose solution to drink; FF + FS = gelatine cube containing fenofibrate at a dose of 100 mg/kg body mass per day + fructose solution to drink. Bst + PW = gelatine cube containing β-sitosterol at a dose of 20 mg/kg body mass per day + plain tap water to drink; Bst + FS = gelatine cube containing β-sitosterol at a dose of 20 mg/kg body mass per day + fructose solution to drink (w/v)

Figure 2 shows the liver histopathology (H and E staining, 40 X magnification) of a representative rat from each of the different treatment groups. The high-fructose diet (PC + FS) caused micro- and macro-vesicular hepatic steatosis. β-sitosterol (Bst + FS) and fenofibrate (FF + FS) prevented the high-fructose diet-induced macro-vesicular hepatic steatosis.

### Non-alcoholic fatty-liver disease frequency

Table 7 depicts the hepatic micro- and macro-vesicular steatosis, hypertrophy, lobular inflammation scores and total NAS of the rats. Hepatic micro- and macro-vesicular steatosis was observed in 100% of the sampled high-fructose diet-fed (PC + FS). β-sitosterol (Bst + FS) and fenofibrate (FF + FS) attenuated the high-fructose diet-induced micro-vesicular steatosis and prevented (*p* = 0.0063; Table 7) macro-vesicular steatosis in 100% of the sampled rats.

**Table 7:** Non-alcoholic fatty liver disease activity score criteria percentage frequency of high-fructose diet-fed female rats

Criteria	Steatosis								Hypertrophy				Inflammation				Total NAS		
	Microvesicular				Macrovesicular														
Score	**0**	**1**	**2**	**3**	**0**	**1**	**2**	**3**	**0**	**1**	**2**	**3**	**0**	**1**	**2**	**3**	**<2**	**3-4**	**≥ 5**
PC + PW	100	0	0	0	100	0	0	0	75	25	0	0	50	50	0	0	100	0	0
PC + FS	0	25	75	0	0	25	50	25	50	25	25	0	0	25	75	0	50	0	50
FF + FS	25	75	0	0	100	0	0	0	75	25	0	0	25	75	0	0	75	25	0
Bst + PW	100	0	0	0	100	0	0	0	100	0	0	0	50	50	0	0	100	0	0
Bst + FS	0	100	0	0	100	0	0	0	75	25	0	0	25	75	0	0	100	0	0

PC + PW = plain gelatine cubes + plain tap water to drink; PC + FS = plain gelatine cubes + 20% (w/v) fructose solution to drink; FF + FS = gelatine cube containing fenofibrate at a dose of 100 mg/kg body mass per day + fructose solution to drink. Bst + PW = gelatine cube containing β-sitosterol at a dose of 20 mg/kg body mass per day + plain tap water to drink; Bst + FS = gelatine cube containing β-sitosterol at a dose of 20 mg/kg body mass per day + fructose solution to drink (w/v);. *n* = 4 per treatment. NAS criteria: steatosis grade scoring 0: 0–5%; 1: 5–33%; 2: 33–66%; 3; > 66%; foci of lobular inflammation scoring 0: none; 1: 1–2; 2: >2. Hypertrophy/ballooning scoring 0: none; 1:1–2; 2: >2. Total NAS score interpretation: <2 = not steatohepatitis; 3–4 = uncertain; ≥ 5 = steatohepatitis

### Renal histology

**Fig. 3:**
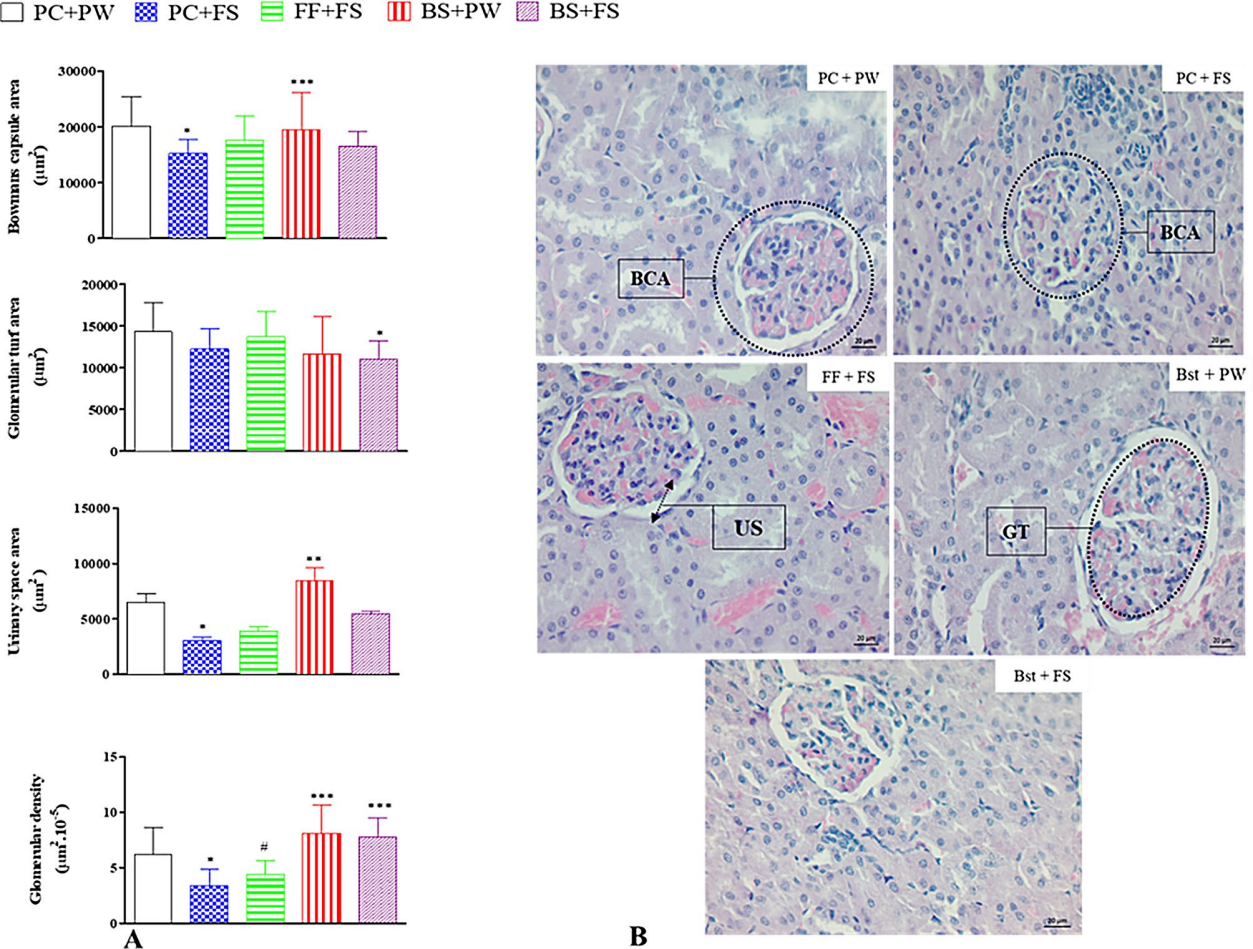
The effect of β-sitosterol on glomerular morphological parameters (**A**). Representative kidney histology (H and E staining, 400 X magnification) (**B**) in high-fructose diet-fed female Sprague-Dawley rats. * *p* < 0.05 when compared to control PC + PW; **p < 0.001 when compared to PC + FS, FF + FS and Bst + FS; *** *p* < 0.0001 when compared to PC + FS; # *p* < 0.05 when compared to Bst + FS and Bst + PW. PC + PW = plain gelatine cubes + plain tap water to drink; PC + FS = plain gelatine cubes + 20% (w/v) fructose solution to drink; FF + FS = gelatine cube containing fenofibrate at a dose of 100 mg/kg body mass per day + 20% (w/v) fructose solution to drink. Bst + PW = gelatine cube containing β-sitosterol at a dose of 20 mg/kg body mass per day + plain tap water to drink; Bst + FS = gelatine cube containing β-sitosterol at a dose of 20 mg/kg body mass per day + 20% (w/v) fructose solution to drink. BCA = Bowman’s capsule area; GT = glomerular tuft; US = Urinary space. Scale bar = 20 μm in the H&E stain sections. Data presented as mean ± SD; *n* = 4 per treatment

Figure 3 shows the effect of β-sitosterol on glomerular morphological parameters (Bowman’s capsule area, glomerular tuft area, urinary space area and glomerular density) and morphologic parameters of renal histology (H and E staining, 400 X magnification) from the different treatment groups. The high-fructose diet decreased glomerular density (*p* = 0.0013 when compared to PC + PW). Moreover, the high-fructose diet reduced (*p* < 0.05) the Bowman’s capsule area and urinary space area when compared to PC + PW. β-sitosterol (Bst + FS) protected (*p* < 0.05; compared to PC + FS) against the high-fructose diet-induced decrease in glomerular density. Fenofibrate (FF + FS) failed to protect (*p* > 0.05) against the high-fructose diet-induced renal histological changes.

## Discussion

In several studies using adult rodents, a high-fructose diet was shown to induce features of metabolic syndrome [26, 27]. We have previously reported that the high-fructose diet animal model used in the current study resulted in increased visceral obesity, hypertriglyceridaemia, and lowered adiponectin levels [28], all features of metabolic syndrome. These characteristics are considered as significant factors in the development of kidney disease and NAFLD. In this context, we decided to investigate the potential renal and liver complications associated with the current model and the potential renoprotective and hepatoprotective effects of orally administrated β-sitosterol in growing female Sprague-Dawley rats mimicking children fed an obesogenic diet. The present study has shown that consumption of 20% fructose diet caused oxidative stress, hepatic steatosis, glomerular structural alterations, and tubular injury. The administration of β-sitosterol protected against the high-fructose diet induced hepatic steatosis, oxidative stress and glomerular structural changes through its antioxidant activity.

The liver is the primary organ in lipogenesis [29]. Long term dietary fructose consumption induces changes in hepatic lipid and carbohydrate metabolism which ultimately causes accumulation and storage of lipids in the liver [30]. Hepatic lipid accumulation was evidenced in the current study, with a 25% more hepatic lipid accretion following the 12 weeks consumption of fructose (Fig. 1). Hepatic lipid accretion, typically, leads to hepatocellular changes associated with non-alcoholic liver disease. However, quantifying the lipid content alone, does not provide detail into the nature of steatosis and impact on hepatic integrity. Therefore, to assess the progression and severity of NAFLD, semi-quantitative non-alcoholic fatty liver disease activity scores (NAS) were used. In our study, the high-fructose diet caused NAFLD as evidenced by micro- and macro-vesicular hepatic steatosis (Fig. 2). Our findings are in conformity with those from other researchers implicating the development of hepatic steatosis following fructose consumption [22, 23]. Orally administered β-sitosterol protected the rats against diet-induced micro- and macro-vesicular hepatic steatosis (Fig. 2). Our results suggest that β-sitosterol could be beneficial against diet-induced steatosis and thus reducing the life-threatening liver-related complications associated with NAFLD. To further assess the hepatoprotective effects of β-sitosterol, our study investigated plasma AST and ALT activities which are considered surrogate biomarkers for liver injury [24, 25]. Previous studies have shown that liver enzymes correlate well with inflammation, and necrosis [26]. The increased inflammation and necrosis lead to increased permeability of the cell membrane [27]. As a result, AST and ALT are released into the bloodstream, causing elevated activities of these enzymes in the plasma. In the current study, all our treatment groups showed no lobular inflammation and necrosis which explains the lack of change in plasma AST and ALT observed between the different groups of rats (Table 2). The liver enzyme results suggest that our interventions neither damaged hepatocytes nor compromised liver function.

Literature evidence suggests that fructose consumption is linked with renal injury [6]. In the small intestine, fructose is absorbed unchanged and then transported to the liver where it is metabolised via fructolysis [31]. Fructolysis is an unregulated process, resulting in the production of intracellular lactate, glucose and fatty acids that enters circulation [32]. Consequently, an increase in fructose intake increases their production and release into circulation, where they are absorbed by variety of cell types including the kidney, thereby contributing to the early onset of renal damage [33, 34]. In this study, histopathological changes in the kidney tissues were recorded as evidence of decreased glomerular density, reduced Bowman’s capsule area and urinary space area following consumption of high-fructose diet (Fig. 3). Glomerular density is the number of glomeruli per area of cortex and a decrease in glomerular density is associated with damage and loss of kidney function [35], renal vasoconstriction [36], and metabolic syndrome [36, 37]. Based in our findings, it can thus be inferred that dietary fructose caused glomeruli disturbance and thus affect the proper functioning of the kidney. Orally administered β-sitosterol for 12 weeks protected against the high-fructose diet-induced decrease in glomerular density (Fig. 3). Our findings suggest that β-sitosterol may have exerted glomeruli protective effects and thus provide beneficial effects against glomeruli changes associated with dietary fructose intake.

Early markers of tubular and glomeruli damage can play an important role in improving kidney function and delaying disease progression [38]. KIM-1 a transmembrane protein serves as a useful biomarker for renal proximal tubule injury facilitating the early diagnosis of the renal disease [14, 39]. The current study showed, increased plasma levels of KIM-1 in high-fructose diet fed rats (Table 4) in comparison to the control rats thus demonstrating evidence of possible tubular damage. Our findings are consistent with previous research that documented that dietary fructose causes renal tubule damage. It has been reported that high-fructose increases uric acid production, resulting in increased production of monocyte chemotactic protein (MCP-1) and reactive oxygen species, all of which contributes to renal tubule damage [17, 31]. Orally administered β-sitosterol was unable to protect against the fructose-induced proximal tubular damage as indicated by elevated plasma KIM-1 concentration (Table 4). Interestingly, treatment with β-sitosterol alone, resulted in elevated plasma KIM-1 concentration (Table 4). Literature trends have shown that KIM-1 can also increase as a result of increased renal proximal tubular epithelial cell regeneration [40] which needs to be further explored in the context of this study.

NGAL serves as an early biomarker of acute renal injury [41]. The fructose-induced tubular injury results were further evidenced by increased in levels of NGAL in the high-fructose fed rats (Table 4). Literature has reported that plasma and urine NGAL concentration are able to predict impending loss of renal function better than serum creatinine [42, 43]. Results of our study collaborate these reports as the consumption of 20% fructose diet for 12 weeks had normal plasma creatinine and urea (Table 3) but with increased NGAL concentration (Table 4). Additionally, it has been suggested that renal failure increases plasma creatinine and urea concentration only when glomerular filtration rate (GFR) is reduced to ~ 50% [44]. The research results observed in this study may therefore suggest that despite the high-fructose diet causing renal tubular injury, it did not reduce GFR below 50% as evidence by normal plasma creatinine and urea concentration. Treatment with β-sitosterol failed to prevent the high-fructose diet increase in NGAL (Table 4). However, it is believed that NGAL has a protective role in case of renal damage, since it contributes to the recovery of the epithelium after injury by increasing in epithelial cell growth [45]. Taken together, the increase in NGAL and KIM-1 in β-sitosterol treated rats might be attributed to the repairment effects. Future studies are warranted to elucidate the molecular mechanism by which this occurs.

The imbalance in reactive oxidative species (ROS) production and antioxidant defense status invariably leads to oxidative stress which is involved in the development of hepatic and kidney damage [45, 46]. One of the primary events during oxidative stress is peroxidative damage to membrane lipids, which can be determined by MDA, their degradation product. Findings from our study revealed that MDA, one of the lipid peroxidation’s by-products, was increased in rats fed a high-fructose diet (Table 5). The activity of anti-oxidant enzymes is important in the protection of cells against ROS-induced cellular damage [46]. We then examined the antioxidant status by determining the rats’ total antioxidant capacity. Our results demonstrated that compared to normal control rats, high fructose-diet rats exhibited reduced TAOC (Table 5). Treatment with β-sitosterol ameliorated oxidative stress by restoring the activities of the total antioxidant levels and suppressing lipid peroxidation in the fructose-induced rats (Table 5). Furthermore, the rats that were fed β-sitosterol alone had an increase in total antioxidant activity. Our findings are consistent with results that have reported antioxidant effects of β-sitosterol [47, 48]. Collectively, our study findings may therefore suggest that the antioxidant activity of β-sitosterol may, in part, be beneficial in lowering the risk of developing oxidative stress-related liver and kidney complications.

## Conclusions

The present study showed that a 20% fructose diet caused NAFL, oxidative stress, tubular and glomerular damage. β-sitosterol effectively protected against the high fructose-induced hepatic lipid accumulation and glomerular damage by decreasing oxidative stress and upregulating the antioxidant defense system.These findings suggest potential prophylactic role for the use of β-sitosterol in the prevention of NAFLD and high-fructose diet induced negative kidney outcomes, hence mitigating the current epidemic MetS-associated NAFLD and renal injury. Our observations also encourage further research to substantiate our findings such as gene and receptor interactions and the developments of potential clinical trials to explore the overall therapeutic potency of β-sitosterol.

## Data Availability

All data produced and analysed in the present study are included in this published paper.

## Abbreviations

NAFL: Non-alcoholic fatty liver
NAFLD: Non-alcoholic fatty liver disease
NASH: Non-alcoholic steatohepatitis
NCDs: Non-communicable chronic diseases
AESC: Animal Ethics Screening Committee
ALT: Alanine aminotransferase
AST: Aspartate aminotransferase
Bst: β-sitosterol
CKD: Chronic kidney disease
DMSO: Dimethyl sulfoxide
FF: Fenofibrate Gelatine cube
FS: Fructose solution
GFR: Glomerular filtration rate
KIM-1: Kidney injury molecule-1
MCP-1: Monocyte chemotactic protein-1
%BM: Mass relative to body mass
NAS: Semi-quantitative NAFLD activity score
NGAL-1: Neutrophil gelatinase-associated lipocalin
PC: Plain gelatine cube
PND: Post-natal
PW: Plain drinking water
ROS: Reactive oxidative species SRC: Standard rat chow
TBA: Thiobarbituric acid
T-AOC: Total antioxidant capacity
